# CXCR5^+^CD8^+^ T Cells Shape Antibody Responses *In Vivo* Following Protein Immunisation and Peripheral Viral Infection

**DOI:** 10.3389/fimmu.2021.626199

**Published:** 2021-07-13

**Authors:** Timona S. Tyllis, Kevin A. Fenix, Todd S. Norton, Ervin E. Kara, Duncan R. McKenzie, Shannon C. David, Mohammed Alsharifi, Di Yu, Shaun R. McColl, Iain Comerford

**Affiliations:** ^1^ Department of Molecular and Biomedical Science, School of Biological Sciences, The University of Adelaide, Adelaide, SA, Australia; ^2^ Diamantina Institute, The University of Queensland, Brisbane, QLD, Australia

**Keywords:** CXCR5^+^CD8^+^ T cells, class switching, immunization, infection, antibody response

## Abstract

Crosstalk between T and B cells is crucial for generating high-affinity, class-switched antibody responses. The roles of CD4^+^ T cells in this process have been well-characterised. In contrast, regulation of antibody responses by CD8^+^ T cells is significantly less defined. CD8^+^ T cells are principally recognised for eliciting cytotoxic responses in peripheral tissues and forming protective memory. However, recent findings have identified a novel population of effector CD8^+^ T cells that co-opt a differentiation program characteristic of CD4^+^ T follicular helper (Tfh) cells, upregulate the chemokine receptor CXCR5 and localise to B cell follicles. While it has been shown that CXCR5^+^CD8^+^ T cells mediate the removal of viral reservoirs in the context of follicular-trophic viral infections and maintain the response to chronic insults by virtue of progenitor/stem-like properties, it is not known if CXCR5^+^CD8^+^ T cells arise during acute peripheral challenges in the absence of follicular infection and whether they influence B cell responses *in vivo* in these settings. Using the ovalbumin-specific T cell receptor transgenic (OT-I) system in an adoptive transfer-immunisation/infection model, this study demonstrates that CXCR5^+^CD8^+^ T cells arise in response to protein immunisation and peripheral viral infection, displaying a follicular-homing phenotype, expression of cell surface molecules associated with Tfh cells and limited cytotoxic potential. Furthermore, studies assessing the B cell response in the presence of OT-I or *Cxcr5^-/-^* OT-I cells revealed that CXCR5^+^CD8^+^ T cells shape the antibody response to protein immunisation and peripheral viral infection, promoting class switching to IgG2c in responding B cells. Overall, the results highlight a novel contribution of CD8^+^ T cells to antibody responses, expanding the functionality of the adaptive immune system.

## Introduction

The generation of high affinity, class-switched antibody responses are important for immune defence against infection and conferring host protection following immunisation. Crosstalk between T and B cells is crucial for successful antibody responses. A subset of CD4^+^ T cells known as T follicular helper (Tfh) cells are primarily responsible for interacting with responding B cells and mediating their selection, survival, affinity maturation and class-switch recombination (1, 2). Tfh cells express the follicular-homing chemokine receptor CXCR5, while concurrently downregulating the T-zone homing receptor CCR7, driving their localisation towards CXCL13-rich follicles where they can interact directly with responding B cells (3–6). Interestingly, other immune cell subsets, including invariant natural killer T cells and CD4^+^ regulatory T cells, have also been reported to co-opt a differentiation program characteristic of the Tfh lineage (7–9). In doing so, they acquire a similar phenotype to Tfh cells, including expression of CXCR5, and migrate to follicles where they regulate B cell responses. Notably, in 2007, a population of CXCR5^+^CD8^+^ T cells with Tfh-like characteristics was identified in human tonsils (10). Subsequent investigations identified CXCR5^+^CD8^+^ T cells as a specific subset that also co-opts the Tfh differentiation pathway and migrates to follicles in response to CXCR5-mediated migratory cues (11–13). In the context of chronic follicular-trophic viral infections, CXCR5^+^CD8^+^ T cells control infection through killing infected cells in the follicular microenvironment (11), eliminating viral reservoirs and maintaining the response against chronic infection (13). Furthermore, CXCR5^+^CD8^+^ T cells have been identified in tumours and tumour draining lymph nodes (14–16). In these settings they have been reported to exhibit stem-like properties, presenting as promising targets for anti-PD-1 therapy and displaying superior anti-tumour functionality compared to their CXCR5^-^ counterparts.

While the cytotoxic functions of CXCR5^+^CD8^+^ T cells have been described in the setting of chronic infection, important questions regarding the context-specific functionality of these cells remain. Specifically, whether CXCR5^+^CD8^+^ T cells participate in humoral immunity requires further investigation. Indeed, *in vitro* studies have demonstrated that CXCR5^+^CD8^+^ T cells can support B cell antibody production (10, 17–19). Additionally, studies exploring settings where CXCR5^+^CD8^+^ T cell differentiation is enhanced have found an associated increase in self-reactive antibody responses, indicating CXCR5^+^CD8^+^ T cells promote autoimmune humoral immunity (20, 21). However, whether CXCR5^+^CD8^+^ T cells participate in physiological humoral immunity *in vivo* is yet to be established. Therefore, in the present study, using models where there is an absence of infection in the follicular microenvironment, we investigated the role of CXCR5^+^CD8^+^ T cells in humoral immune responses *in vivo* in settings where there is no necessity for cytotoxic function within the follicular microenvironment. We show that CXCR5^+^CD8^+^ T cells develop in response to protein immunisation and influenza A virus (IAV) infection, displaying a follicular-homing phenotype and surface expression profile similar to Tfh cells, as well as exhibiting diminished cytotoxic potential. In addition, we demonstrate that these cells shape antibody responses, promoting IgG2c class switching in responding B cells in these settings. Taken together, these data show that in addition to their previously described cytotoxic functions in response to infections in the follicular microenvironment, CXCR5^+^CD8^+^ T cells also shape antibody responses *in vivo*.

## Materials and Methods

### Mice

C57BL/6 and Ly5.1 mice were purchased from the Animal Resource Centre (WA, Australia). OT-I mice were purchased from the Walter and Eliza Hall animal facility (Kew: VIC, Australia) and crossed with Ly5.1 mice at the University of Adelaide animal facility to obtain OT-I CD45.1/CD45.2 (CD45.1/2) mice. CXCR5-deficient (*Cxcr5^-/-^*) mice were bred at the University of Adelaide animal facility and crossed to OT-I mice to generate *Cxcr5^-/-^* OT-I mice. Transgene status of OT-I and *Cxcr5^-/-^* OT-I mice was determined by flow cytometric analysis of TCR Vα2 expression. Interferon-gamma-deficient (B6*.Ifng^-/-^*) mice were obtained from the Queensland Institute of Medical Research (QIMR) Berghofer animal facility. Age matched, female mice at 6-16 weeks of age were used in experiments. Experiments were conducted under approval from the University of Adelaide Animal Ethics Committee.

### Immunisations, Infections, Adoptive Cell Transfers, and Treatments

Ovalbumin precipitated in alum (OVA/alum) was prepared by diluting a 1 mg/mL solution of ovalbumin (Sigma-Aldrich) in PBS to 0.5 mg/mL in α-minimal essential media (αMEM, Gibco) supplemented with 25 mM HEPES (Gibco). An equal volume of a 10% w/v solution of aluminium potassium sulphate (BDH chemicals Ltd) prepared in MilliQ water was then added and the resulting solution was adjusted to pH of 6.5 with 1 M sodium hydroxide and 1 M hydrochloric acid. The solution was then washed extensively with PBS by centrifugation at 500g for 10min at 4°C. The precipitated OVA/alum was resuspended at 0.5 mg/mL in PBS and 200 µL (100 µg) was injected into mice intraperitoneally (i.p). The HKx31 (x31) and HKx31-OVA (x31-OVA) influenza A viruses (IAVs) have been described previously (22). Virus stocks for infections were grown in chicken eggs. For IAV infections, mice were anesthetised with 60 µg/g of pentabarbitone (ilium) in PBS i.p and a dose of 10 TCID50 of x31 or x31-OVA diluted in PBS was administered intranasally in a volume of 32 µL. OT-I and *Cxcr5^-/-^* OT-I cells were negatively isolated from spleens using the EasySep™ Mouse Naïve CD8^+^ T Cell Isolation Kit (STEMCELL Technologies), as per the manufactures guidelines, at routinely >90% purity. For experiments in the OVA/alum model, 2x10^5^ OT-I cells were transferred i.v. in 200 µL of PBS a day prior to immunisation, either from WT OT-I or *Cxcr5^-/-^* OT-I mice. This was the same for the x31-OVA model except 1x10^4^ OT-I cells were transferred instead. To deplete CD8^+^ T cells, mice were treated with 100 µg of αCD8β (clone 53-5.8, Bio X cell) in 200 µL PBS i.p 4 days prior to IAV infection and on day 4 (and again on day 12 where required) post-infection. Control mice were treated with an equivalent quantity of rat γ-globulin (Rockland).

### Flow Cytometry

Single-cell suspensions were prepared by pressing spleens and mediastinal lymph nodes through 70µm nylon filters (FALCON). Red blood cells were lysed by incubating spleen cell suspensions in red cell lysis buffer (139.5 mM NH4Cl, 17 mM Tris-HCl, pH 7.2) for 5 minutes at 37°C. Cells were stained in 96-well round-bottom trays (Corning) at 2x10^6^ cells/well. For intracellular cytokine staining, cells were incubated in complete IMDM containing 20 ng/mL phorbol 12-myristate 13-acetate (PMA, Sigma-Aldrich) and 1 µM Ionomycin (Life Technologies) for a total of 4 hrs at 37°C with addition of 1/1500 GolgiPlug (Brefeldin A, BD) and 1/1500 GolgiStop (containing monensin, BD) for the final 3hrs, before staining. OT-I cells were stimulated with SIINFEKL peptide (InvivoGen) at 1 µg/mL in IMDM for 4hrs in the presence of GolgiPlug and GlogiStop for the final 3hrs. Cells were washed with PBS and stained with Near Infrared Fixable Viability Dye (Life Technologies) diluted 1/1,000 in PBS for 15 min at room temperature (RT). Subsequently, cells were washed in FACS buffer (PBS containing 1% bovine serum albumin and 0.04% sodium azide) then blocked with 200 μg/mL mouse γ-globulin (Rockland) for 10-15 minutes at RT. For staining immunoglobulin isotypes, cells were blocked with 200 μg/mL rat γ-globulin (Rockland) for 10-15 minutes at RT. The following steps were performed at 4°C unless otherwise stated. For CXCR5 staining, cells were incubated with purified α CXCR5 (BD, clone 2G8) for 1 h, washed with FACS buffer then incubated with fluorophore-labelled secondary antibody in the presence of mγg (200 μg/mL) and normal mouse serum (NMS) (1%) for 30-40 min before washing in FACS buffer and blocking with 200 μg/mL rat γ-globulin (Rockland) for 10-15 minutes. Cells were then stained with fluorophore-labelled and biotinylated antibodies (BD; CD8 (53.6-7), CD4 (GK1.5), CD44 (IM7), CD45.1 (A20), CD45.2 (104), B220 (RA3-6B2), KLRG1 (2F1), CD127 (SB/199), Tim-3 (5D12/TIM-3), CD138 (281-2), IgD (11-26c.2a): eBioscience; ICOS (C398.4A), PD-1 (J43), BTLA (8F4): BioLegend; T/B Cell Act. Marker (GL7), Ly108 (330-AJ)) for 20 minutes and washed with PBS containing 0.04% sodium azide. For staining intracellular cytokines and immunoglobulin isotypes, cells were incubated in Cytofix/Cytoperm (BD) for 25-30 minutes, washed in Permwash (BD) and incubated with fluorophore-labelled and biotinylated antibodies (BD; IFNγ (XMG1.2), TNFα (MP6-XT22), GzmB (GB11), IgG2a/c (5.7), eBioscience; IgM (eB121-15F9), IgG1 (M1-14D12)) for 25-30 minutes in Permwash. For transcription factor staining, cells were incubated in 100 µL of freshly prepared FOXP3 Fixation/Permeabilisation Solution (Invitrogen) for 25-30 minutes at RT in the dark, before washing in FOXP3 Permeabilisation Buffer (Invitrogen) and staining with fluorophore-labelled antibodies [BD; TCF-1 (S33-966)] in Permeabilisation Buffer for 25-30 minutes. For detection of biotinylated antibodies, cells were incubated with fluorophore-labelled streptavidin for 20 minutes. After washing in PBS with 0.04% sodium azide, cells were resuspended in PBS with 1% paraformaldehyde and stored at 4°C in the dark. Acquisition was performed on a BD LSR II, BD FACSAria or BD LSR Fortessa and analysed with FlowJo Software (BD).

### Enzyme-Linked Immunosorbent Assays (ELISAs)

For analysis of x31-specific antibodies in the serum of mice infected with x31/x31-OVA, concentrated x31 (~70x10^6^ TCID50) was diluted 1/200 in 0.1 M sodium bicarbonate and incubated overnight at RT in 96-well flat-bottom EIA/RIA high-binding plates (Corning). The plates were then washed with PBS containing 0.05% Tween (AMRESCO) (PBS/Tween) and blocked with PBS containing 2% skim milk for 2 h at RT. Plates were washed with PBS/Tween and then incubated with serum dilutions prepared in PBS for 2 h at RT before washing with PBS/Tween and incubation with anti-mouse isotype-specific horseradish peroxidase (HRP) conjugated antibodies (SouthernBiotech; IgM, IgG, IgG1, IgG2c, IgG3) diluted in PBS containing 2% skim milk for 2 h at RT. After washing with PBS/Tween, the plates were developed by adding 1xTMB substrate solution (Invitrogen) for 10-20 minutes in the dark. The reaction was stopped by addition of 1 M orthophosphoric acid and absorbance was measured at 450 nM on a Biotrack II plate reader (Amersham BioScience).

### *Ex Vivo* Cytotoxicity Assay

E0771-mCherry and E0771-GFP-OVA cells were grown in DMEM with 10% FCS and 100 U/mL Penicillin/Streptomycin, harvested and stained with Cell Proliferation Dye eFlour670 (eBioscience) and carboxyflourescien succinimidyl ester (CFSE, Molecular Probes) respectively, according to the manufacture’s guidelines. Subsequent to dye staining, cells were washed twice in complete IMDM and resuspended to a concentration of 2.0x10^5^ cells/mL in complete IMDM. Equal volumes of the dye-labelled E0771-mCherry and E0771-GFP-OVA cells were mixed and 100 µL of the resulting cell suspension was then distributed to the appropriate wells of a 96-well flat-bottom plate (20,000 cells/well total), followed by incubation for 4 hours at 37°C in 5% CO_2_. Effector CXCR5^+^ and CXCR5^-^ OT-I cells (pre-gated on live, CD4^-^B220^-^CD8^+^TCRβ5.1^+^CD44^+^ cells) were FACS-sorted from the spleens of OT-I mice immunised seven days earlier with OVA/alum i.p. Sorted OT-I cells were incubated with E0771 cells at effector cell to target cell ratios of 0.5:1, 1:1 and 5:1 for 12 hours at 37°C in 5% CO_2_. The supernatant was then transferred to a new 96 well V-bottom plate. Wells were then washed in 50 µL of 1X PBS that was pooled with the supernatant. Adherent E0771 cells were harvested by incubation in 50 µL of trypsin/EDTA for 5 minutes at room temperature. The reaction was neutralised with 100 µL of supernatant obtain previously and cells were pooled into the 96 well V-bottom plate. Cells were then subjected to flow cytometric analysis to assess killing of E0771 target cells.

To quantify cytotoxic activity, the following formulae were utilised:

$$\begin{array}{c} \%\;\text{Specific}\;\text{killing}=[\%\;\text{CFS}{\text{E}}^{+}\;\text{E}0771\;\text{cells}\;\text{that}\;\text{were}\;\text{positive}\;\text{for}\;\text{LIVE}/\text{DEAD}\;\text{viability}\;\text{dye}\;\text{staining}]\\ {}[\%\;670^{+}\text{E}0771\;\text{cells}\;\text{that}\;\text{were}\;\text{positive}\;\text{for}\;\text{LIVE/DEAD}\;\text{viability}\;\text{dye}\;\text{staining}]. \end{array}$$

### Statistics

Data were analysed in GraphPad Prism 8 & 9. Statistical tests were two-sided and applied as detailed in the figure legends. *p<0.05, **p<0.01, ***p<0.001, ****p<0.0001.

## Results

**Figure 1:**
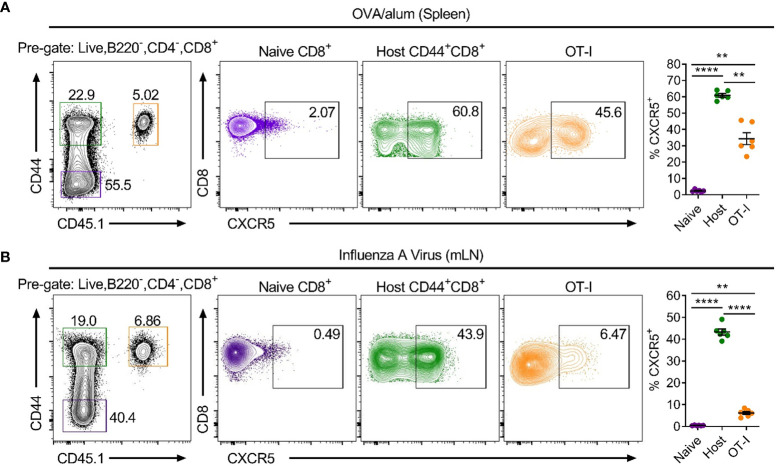
CXCR5^+^CD8^+^ T cells are generated in acute settings in the absence of follicular infection following protein immunisation and IAV challenge. Isolated, congenically marked (CD45.1/2) OT-I cells were transferred i.v into C57BL/6 mice that were then **(A)** immunised with OVA/alum intraperitoneally or **(B)** infected with x31-OVA intranasally. Flow cytometry of **(A)** spleen cells on day 7 post-immunisation with OVA/alum and **(B)** mLN cells on day 8 post-infection with x31-OVA, assessing CXCR5 expression on the indicated cell populations. Data are representative of at least three independent experiments with at least 4 mice. Data were analysed by a repeated measures one-way ANOVA. Mean ± SEM. **p < 0.01, ****p < 0.0001.

To date, CXCR5^+^CD8^+^ T cells have only been shown in chronic infection, cancer and autoimmunity, all settings where there is antigen persistence. To determine if CXCR5^+^CD8^+^ T cells are also generated in response to acute challenges that do not establish chronic infection in the follicular microenvironment, MHC class I-restricted, ovalbumin-specific, CD8^+^ T cells (OT-I cells) were utilised in the context of adoptive transfer-immunisation/infection strategies. Isolated naïve OT-I cells were transferred intravenously into WT C57Bl/6 mice that were subsequently immunised intraperitoneally with ovalbumin precipitated in alum (OVA/alum) or infected intranasally with a recombinant x31 IAV strain expressing the ovalbumin-derived SIINFEKL peptide (x31-OVA). Flow cytometry assessing the response to OVA/alum in the spleen 7 days post-immunisation and x31-OVA in the mediastinal lymph node (mLN) 8 days post-infection, demonstrated that ~60% and ~45% of endogenous CD44^hi^CD8^+^ T cells expressed CXCR5, respectively, compared to naïve CD44^lo^CD8^+^ T cells which displayed minimal positivity for CXCR5 (**Figures 1A, B**). Furthermore, analysis of the antigen-specific OT-I cells revealed that ~45% and ~6.5% were expressing CXCR5 in response to OVA/alum and x31-OVA, respectively (**Figures 1A, B**). These results demonstrate that CXCR5^+^CD8^+^ T cells arise in acute responses in the absence of infection in the follicular microenvironment following protein immunisation and peripheral viral infection.

**Figure 2:**
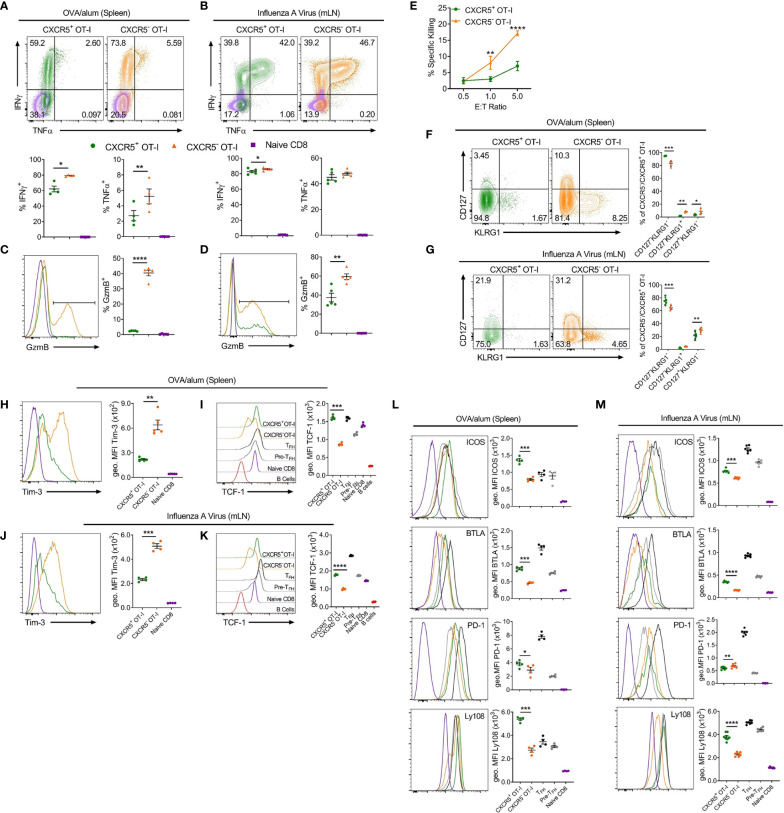
CXCR5^+^CD8^+^ T cells generated in response to OVA/alum and IAV are distinct from their CXCR5^-^ counterparts. Isolated congenically marked (CD45.1/2) OT-I cells were transferred i.v into C57BL/6 mice that were immunised with OVA/alum i.p or infected with x31-OVA i.n the following day. On day 7 post-immunisation and day 8 post-infection, the spleens (OVA/alum) and mLNs (x31-OVA) were harvested and analysed by flow cytometry. **(A, B)** IFNγ and TNFα expression by CXCR5^+^ and CXCR5^-^ OT-I cells and naïve CD8^+^ T cells following **(A)** OVA/alum and **(B)** x31-OVA. **(C, D)** Granzyme B (GzmB) expression by CXCR5^+^ and CXCR5^-^ OT-I cells and naïve CD8^+^ T cells following **(C)** OVA/alum and **(D)** x31-OVA. **(E)** *Ex vivo* cytotoxicity assay assessing the specific killing of CXCR5^+^ and CXCR5^-^ OT-I cells FACS-sorted from the spleens of mice immunised i.p with OVA/alum 7 days prior. **(F, G)** CD127 and KLRG1 expression by CXCR5^+^ and CXCR5^-^ OT-I cells following **(F)** OVA/alum and **(G)** x31-OVA. **(H, J)** Tim-3 expression by CXCR5^+^ and CXCR5^-^ OT-I cells and naïve CD8^+^ T cells following **(H)** OVA/alum and **(J)** x31-OVA. **(I, K)** TCF-1 expression by CXCR5^+^ and CXCR5^-^ OT-I cells, TFH, Pre-TFH, Naïve CD8^+^ T cells and B220^+^ B cells following **(I)** OVA/alum and **(K)** x31-OVA. **(L, M)** Expression of ICOS, BTLA, PD-1 and Ly108 by CXCR5^+^ and CXCR5^-^ OT-Is, TFH, Pre-TFH and Naïve CD8^+^ T cells following **(L)** OVA/alum and **(M)** x31-OVA. Refer to **Figure 1** for gating of CXCR5^+^ and CXCR5^-^ OT-I cells and Naïve CD8^+^ T cells. Refer to **Supplementary Figure 1A** for gating of TFH and Pre-TFH populations. **(A–D, F–M)** Data are representative of at least two independent experiments with at least 4 mice. **(E)** Data are representative of two independent experiments with 3 mice. **(A–D, F–M)** Data were analysed by paired t-tests or **(E)** two-way ANOVA with Bonferroni’s multiple comparison test to compare CXCR5^+^ and CXCR5^-^ OT-I cells. **(A–D, F–M)** Mean ± SEM or **(E)** Mean ± SD. *p < 0.05, **p < 0.01, ***p < 0.001, ****p < 0.0001.

To explore possible functions of CXCR5^+^CD8^+^ T cells identified in these settings, activated CD44^hi^ CXCR5^+^ and CXCR5^-^ OT-I cells were compared for expression of key cytokines and cell surface molecules (**Figures 2A–M**). Intracellular cytokine staining demonstrated that CXCR5^+^CD8^+^ T cells expressed less IFNγ than CXCR5^-^CD8^+^ T cells in response to OVA/alum and x31-OVA (**Figures 2A, B**), although this small difference is unlikely to be physiologically relevant. CXCR5^+^CD8^+^ T cells produced less TNFα than their CXCR5^-^ counterparts in response to OVA/alum while CXCR5^+^ and CXCR5^-^CD8^+^ T cells expressed similar levels of TNFα in response to x31-OVA (**Figures 2A, B**). In both models, CXCR5^+^CD8^+^ T cells had significantly reduced levels of granzyme B (GzmB) than CXCR5^-^CD8^+^ T cells (**Figures 2C, D**). Furthermore, in an *ex vivo* cytotoxicity assay, CXCR5^+^CD8^+^ T cells generated in response to OVA/alum were substantially less potent cytotoxic effector cells than their CXCR5^-^ counterparts (**Figure 2E**). In both models, it was apparent that CXCR5^+^CD8^+^ T cells are enriched for cells with a KLRG1^-^CD127^-^ surface phenotype (**Figures 2F, G**), which in the OVA/alum model are predominantly early effector cells. In addition, CXCR5^+^CD8^+^ T cells in both models also displayed a Tim-3^lo^TCF-1^hi^ signature indicative of a precursor-exhausted phenotype (**Figures 2H–K**), similar to the profile of CXCR5^+^CD8^+^ T cells identified in LCMV infection (23). Further profiling identified that CXCR5^+^CD8^+^ T cells express higher levels of Tfh-associated cell surface molecules, including ICOS, BTLA and Ly108 in response to OVA/alum and x31-OVA than that present on CXCR5^-^CD8^+^ T cells (**Figures 2L, M**). PD-1 levels were slightly higher on CXCR5^+^CD8^+^ T cells in OVA/alum while they were slightly lower between CXCR5^+^ and CXCR5^-^ CD8^+^ T cells in x31-OVA (**Figures 2L, M**). Overall, these results indicate that CXCR5^+^CD8^+^ T cells are generated in acute settings in response to protein immunisation and IAV and are phenotypically distinct from their CXCR5^-^ counterparts, highlighting the potential for differing functionalities.

**Figure 3:**
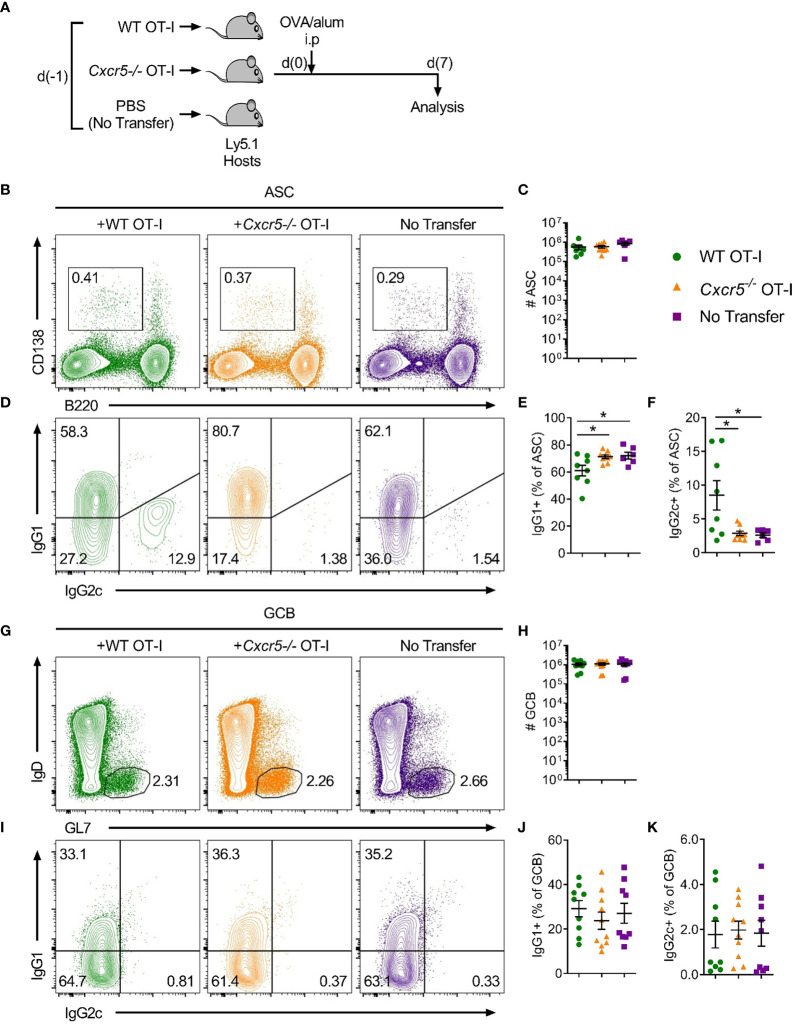
CD8^+^ T cells require CXCR5 expression to skew class switching from IgG1 to IgG2c in ASCs in response to OVA/alum. Congenically marked WT (CD45.1/2) or *Cxcr5* ^-/-^ (CD45.2) OT-I cells (or PBS only for no transfer controls) were transferred i.v into separate B6.Ly5.1 (CD45.1) mice that were subsequently immunised with OVA/alum i.p. and harvested on day 7 post-immunisation for analysis. **(A)** Schematic outline of the experiment. **(B)** Gating strategy used for identification of CD138^+^B220^int^ ASCs in spleens. Pre-gated on live, single cells. **(C)** Total number of ASCs. **(D)** Analysis of intracellular IgG1 and IgG2c in IgM^-^ ASCs. **(E, F)** Frequency of **(E)** IgG1^+^ and **(F)** IgG2c^+^ ASCs among total ASCs. **(G)** Gating strategy used for identification of B220^+^IgD^-^GL7^+^ GCB cells in spleens. Pre-gated on live, single, B220^+^CD138^-^ cells. **(H)** Total number of GCB cells. **(I)** Analysis of intracellular IgG1 and IgG2c in total GCB cells. **(J, K)** Frequency of **(J)** IgG1^+^ and **(K)** IgG2c^+^ GCB cells among total GCB cells. **(C, E, F, H, J, K)** Data are pooled from two independent experiments with a total of 6-10 mice per group. Data were analysed by ordinary one-way ANOVA. Mean ± SEM. *p < 0.05.

Together, the apparent follicular-homing characteristics, Tfh-like surface phenotype and reduced cytotoxic potential led us to hypothesize that CXCR5^+^CD8^+^ T cells could support B cell responses *in vivo*. Interestingly, a previous study in the OVA/alum immunisation model demonstrated that CD8^+^ T cell-derived IFNγ skewed switching patterns in responding B cells from IgG1 to IgG2a (24). To determine whether CXCR5^+^CD8^+^ T cells influence class switching in B cells in response to OVA/alum, wild type (WT) or *Cxcr5^-/-^* OT-I cells were transferred into separate hosts which were then immunised with OVA/alum and the response in the spleen was assessed 7 days later (**Figure 3A**). Transfer of WT or *Cxcr5^-/-^* OT-I cells did not affect IFNγ production in endogenous CD4^+^ T cells and did not modulate the frequency of Tfh or Th1 cells (**Supplementary Figures 1B–F**). Within the B cell compartment, transfer of OT-I cells did not influence the numbers of CD138^+^B220^int^ antibody secreting cells (ASCs) (**Figure 3C**) or B220^+^IgD^-^GL7^+^ germinal centre B (GCB) cells (**Figure 3H**). However, an OT-I cell-dependent, ASC-restricted, induction of IgG2c class switching, which appeared to be at the expense of class switching to IgG1, was apparent that was dependent on *Cxcr5* expression by the transferred OT-I cells (**Figures 3B–F**). There was no apparent role for OT-I cells in regulating IgG2c class switching in GCB cells (**Figures 3G–K**). Importantly, loss of CXCR5 on OT-I cells did not greatly affect their capacity to respond to OVA/alum immunization and these cells efficiently produced IFNγ (**Supplementary Figures 1G, H**). Collectively, these data demonstrate that CXCR5^+^CD8^+^ T cells promote IgG2c class switching in ASCs in response to protein immunisation, likely through local delivery of IFNγ to responding B cells.

**Figure 4:**
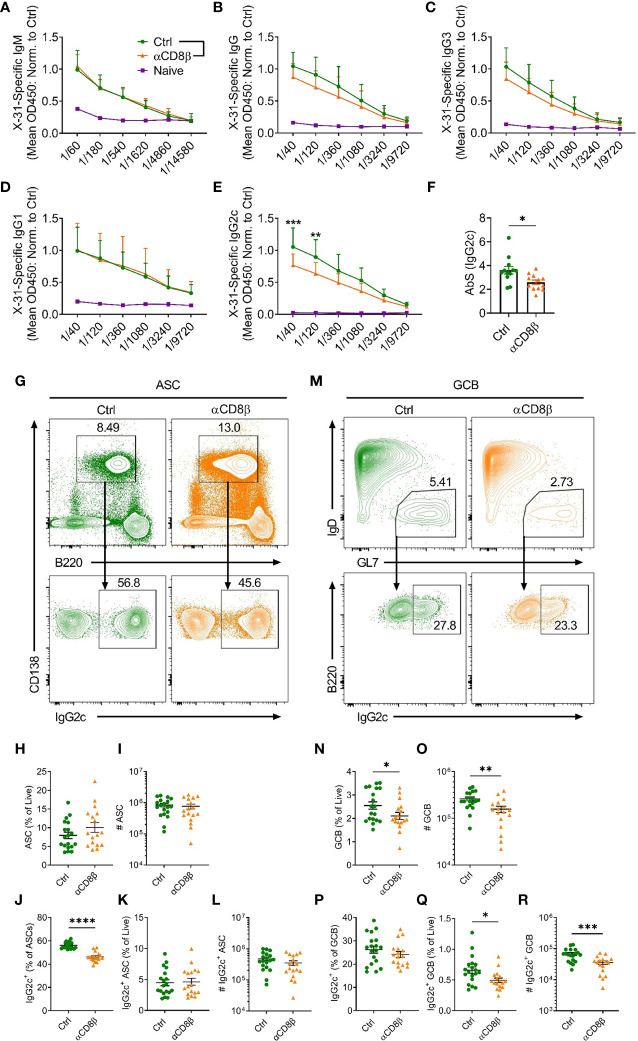
CD8^+^ T cells support induction of IgG2c responses against IAV. C57BL/6 mice were treated i.p. with either αCD8β or control antibody 4 days before and on day 4 after i.n infection with x31 and harvested on day 8 post-infection. Levels of serum x31-specific **(A)** IgM, **(B)** IgG, **(C)** IgG3, **(D)** IgG1 and **(E)** IgG2c assessed by ELISA. **(F)** Absorption summation (AbS) analysis of the x31-specific IgG2c ELISA data in **(E)**. AbS is determined by adding the absorbance values from all dilutions to obtain a single value for each biological replicate (25). **(G)** Gating strategy used for identification of CD138^+^B220^int^ ASCs and IgG2c-switching in ASCs in the mLN. Cells in the top panel were pre-gated on live, single cells. **(H, I)** Quantification of the ASC response by **(H)** frequency and **(I)** number. **(J–L)** Analysis of the IgG2c^+^ ASC response by **(J)** proportion of total ASCs, **(K)** frequency of total live cells and **(L)** number. **(M)** Gating strategy used for identification of B220^+^IgD^-^GL7^+^ GCB cells and IgG2c-switching in GCB cells in the mLN. Cells in the top panel were pre-gated on live, B220^+^CD138^-^ cells. **(N, O)** Quantification of the GCB cell response by **(N)** frequency and **(O)** number. **(P–R)** Analysis of the IgG2c^+^ GCB cell response by **(P)** proportion of total GCB cells, **(Q)** frequency of total live cells and **(R)** number. **(A–F)** Data are pooled from two independent experiments with a total of 12 mice per group (Ctrl and αCD8β) or 3 mice (Naïve) and the absorbance values for the ELISA data have been normalised to the average of the Ctrl group for each independent experiment prior to pooling the data. **(H–L, N–R)** Data are pooled from three independent experiments with 17-19 mice per group. **(A–E)** Data were analysed by two-way ANOVA with Bonferroni’s multiple comparison test or **(F, H–L, N–R)** unpaired t-tests. **(F, H–L, N–R)** Mean ± SEM or **(A–E)** Mean + SD. *p < 0.05, **p < 0.01, ***p < 0.001, ****p < 0.0001.

To extend the finding that CXCR5^+^CD8^+^ T cells promote IgG2c class switching in ASCs in the absence of chronic follicular infection, we determined whether CXCR5^+^CD8^+^ T cells could support B cell responses to IAV infection. Prior to specifically investigating the role of CXCR5^+^CD8^+^ T cells, we assessed the overall contribution of CD8^+^ T cells to the humoral immune response against IAV by depleting CD8^+^ T cells with an antibody against CD8β. Analysis of serum at day 8 post IAV infection revealed that mice depleted of CD8^+^ T cells had similar levels of x31-specific IgM (**Figure 4A**) and IgG1 (**Figure 4D**), slightly lower levels of x31-specific IgG (**Figure 4B**) and IgG3 (**Figure 4C**), and significantly reduced levels of x31-specific IgG2c (**Figures 4E, F**), when compared to controls. This result was paralleled by a reduction in the proportion of IgG2c class switched ASCs in the mLN in CD8β-depleted mice (**Figures 4G–J**). While the frequency of total ASCs was slightly increased in CD8β-depleted mice (**Figure 4H**), as removal of the CD8^+^ T cell compartment affects the relative frequencies of the remaining cell populations, the total number of ASC was unchanged (**Figure 4I**). With regard to the cellularity of the IgG2c-switched ASC response, the frequency of IgG2c-switched ASCs among live cells was unchanged between the two groups (**Figure 4K**), while the reduced switching to IgG2c in the ASC compartment led to an average ~25% decrease in the total number of IgG2c-switched ASCs in CD8β-depleted mice (**Figure 4L**, Mean (Ctrl) = 472152, Mean (αCD8β) = 341128), although this was not statistically significant. Interestingly, the GCB cell response was significantly impaired in CD8β-depleted mice (**Figures 4M–O**), indicating CD8^+^ T cells support optimal induction of the GCB cell response to IAV infection. This in turn lead to a significant reduction in the cellularity of the IgG2c-switched GCB cell response in CD8β-depleted mice (**Figures 4Q, R**), although the frequency of IgG2c class switching within the GCB cell compartment was unchanged (**Figure 4P**). Further assessment of the humoral response at day 14 post IAV infection revealed that x31-specific IgG2c levels in the serum of CD8β-depleted mice recovered to that of the controls (**Supplementary Figure 1I**), while the cellularity of the GCB cell response had also equilibrated between the two groups (**Supplementary Figures 1J, K**). Overall, we conclude that CD8^+^ T cells are required for optimal induction of the early extrafollicular wave of IgG2c in the humoral immune response against IAV.

**Figure 5:**
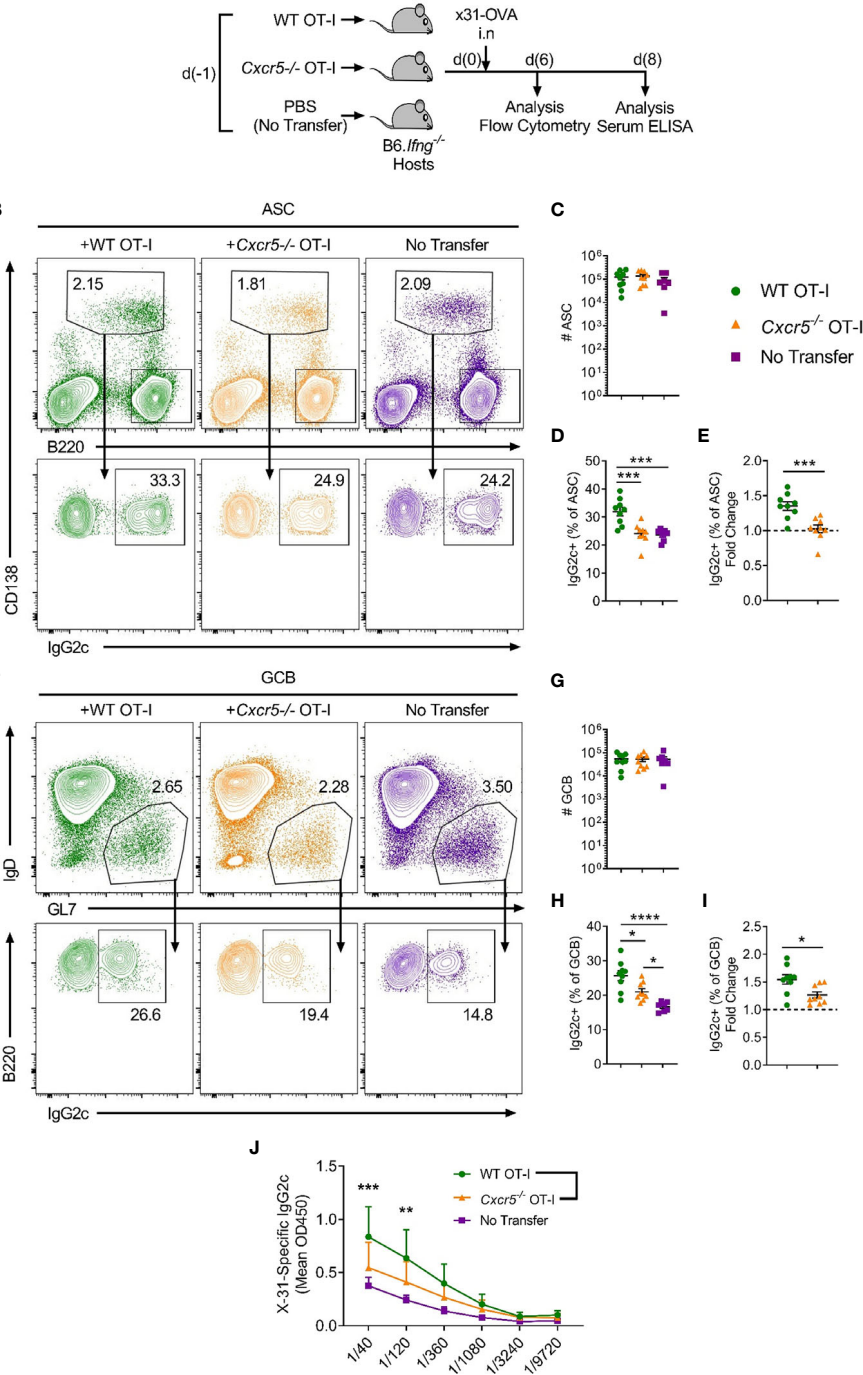
CXCR5^+^CD8^+^ T cells promote class switching to IgG2c in responding B cells following IAV infection. WT or *Cxcr5* ^-/-^ OT-I cells (or PBS only for no transfer controls) were transferred i.v. into separate B6.*Ifng^-/-^* mice that were then infected with x31-OVA i.n. **(A)** Schematic outline of the experiment. **(B)** Gating strategy used for identification of CD138^+^B220^int^ ASCs and IgG2c-switching in ASCs in the mLN. Cells in the top panel were pre-gated on live, single cells. **(C)** Quantification of ASC number. **(D)** Frequency of IgG2c^+^ ASCs among total ASCs. **(E)** Fold change in IgG2c-induction relative to the no transfer control (dashed line). **(F)** Gating strategy used for identification of B220^+^IgD^-^GL7^+^ GCB cells and IgG2c-switching in GCB cells in the mLN. Cells in the top panel were pre-gated on live, single, B220^+^CD138^-^ cells. **(G)** Quantification of GCB cell number. **(H)** Frequency of IgG2c^+^ GCB cells among total GCB cells. **(I)** Fold change in IgG2c-induction relative to the no transfer control (dashed line). **(J)** Levels of serum x31-specific IgG2c on day 8 post-infection assessed by ELISA. **(C–E, G–I)** Data are pooled from two independent experiments with a total of 7-9 mice group or **(J)** representative of two independent experiments with 4-5 mice per group. **(C–E, G–I)** Data were analysed by ordinary one-way ANOVA or **(J)** two-way ANOVA with Bonferroni’s multiple comparison test. **(C–E, G–I)** Mean ± SEM or **(J)** Mean + SD. *p < 0.05, **p < 0.01, ***p < 0.001, ****p < 0.0001.

Notably, IgG2c is the predominant isotype produced in response to viral infection (26) and it is considered a highly effective anti-pathogen isotype (27, 28). Class switching to IgG2c is induced by IFNγ (29) and requires the downstream transcription factor T-bet (30). While the contribution of follicular CD4^+^ T cell-derived IFNγ to IgG2c class switching in responding B cells is well-recognised (31), other cellular sources of IFNγ have also been reported to promote IgG2c class switching, including γδ T (32) cells, NKT cells (33) and CD4^+^ T-helper 1 cells (34). Thus, to specifically investigate a role for CXCR5^+^CD8^+^ T cells in mediating IgG2c class switching in response to IAV, we transferred WT or *Cxcr5^-/-^* OT-I cells into B6.*Ifng^-/-^* mice, infected the recipients with x31-OVA and harvested the mLNs on day 6 post-infection for flow cytometric analyses (**Figure 5A**). While there was a basal level of IFNγ-independent IgG2c class switching in the no-transfer controls, transfer of OT-I cells substantially enhanced class switching to IgG2c in ASCs (**Figures 5B–E**). However, this effect was not observed when *Cxcr5^-/-^* OT-I cells were transferred. Interestingly, in this setting, transfer of WT OT-I cells also significantly enhanced IgG2c class switching in the GCB cell compartment on day 6 post-infection, an effect that was also dependent on CXCR5 expression by OT-I cells (**Figures 5F–I**). This result indicated that CXCR5^+^CD8^+^ T cells can promote IgG2c class switching in the GCB cell compartment when the ability to produce IFNγ was restricted to adoptively transferred CD8^+^ T cells, as is the case in B6.*Ifng^-/-^* recipients. Importantly, there were no differences in IFNγ production between WT and *Cxcr5^-/-^* OT-I cells (**Supplementary Figures 1L–N**), indicating that CXCR5-mediated migratory cues were required for OT-I cells to shape class switching in responding B cells, likely through local delivery of IFNγ to activated B cells. Using the same experimental strategy, analysis of x31-specific IgG2c in the serum on day 8 post-infection revealed that CXCR5 expression in transferred OT-I cells was required for optimal induction of x31-specific IgG2c (**Figure 5J**). Overall, these results demonstrate that CXCR5^+^CD8^+^ T cells shape the humoral response to peripheral viral infection, promoting IgG2c class switching in response to IAV.

## Discussion

CXCR5^+^CD8^+^ T cells have been identified in multiple settings, including tonsils (10, 19), chronic infections [LCMV (11–13), SIV (35, 36), HIV (11, 37), HBV (17, 38)], cancers [lymphoma (11, 18, 39), colorectal (16, 40), thyroid (41)] and autoimmunity (20, 21, 42). In the present study, we further address questions regarding the context-specific generation of CXCR5^+^CD8^+^ T cells, demonstrating that, in addition to their existence in response to chronic stimuli, these cells also arise in acute responses to protein immunisation and peripheral viral infection where there is an absence of infection in the follicular microenvironment. In these settings, CXCR5^+^CD8^+^ T cells exhibit limited cytotoxic potential, display a Tim-3^lo^TCF-1^hi^ signature, indicative of a progenitor-like, precursor-exhausted CD8^+^ T cell phenotype, express Tfh-associated molecules, suggesting they co-opt the follicular differentiation program, and shape class switching in responding B cells *in vivo*, promoting class switching to IgG2c.

Antigen-specific CD8^+^ T (OT-I) cells upregulated CXCR5 in response to protein immunisation and IAV. However, the proportion of host CD44^hi^CD8^+^ T cells expressing CXCR5 was significantly higher than that of the OT-I cells in both models. Additionally, we have also observed CD44^hi^CXCR5^+^CD8^+^ T cells in unchallenged mice (data not shown). Therefore, it is likely that the host CD44^hi^CXCR5^+^CD8^+^ T cells represent endogenous memory and virtual memory CD8^+^ T cells, of which we and others have observed to express CXCR5 [(43) & data not shown]. While CXCR5^+^CD8^+^ T (OT-I) cells were generated in both models, the frequency of OT-I cells expressing CXCR5 was greater in response to protein immunisation compared to IAV. This is likely due to differences in the inflammatory milieu induced by protein immunisation compared with that of a complex pathogen in IAV. In support of this, CXCR5^+^CD8^+^ T cells generated in response to the more inflammatory IAV express more IFNγ, TNFα and GzmB compared to those generated in response to the less inflammatory protein immunisation. Indeed, the proinflammatory cytokine IL-2, which is abundantly expressed in response to IAV, has been identified in recent studies as a negative regulator of CXCR5^+^CD8^+^ T cell generation. Interrupting the IL-2 signalling pathway at various levels through deficiency in IL-2 (20), STAT5 (21) and Blimp1 (11) increases differentiation of CXCR5^+^CD8^+^ T cells. Furthermore, IL-2 has been shown to supress the follicular differentiation pathway and generation of CXCR5^+^CD4^+^ T follicular helper (44) and follicular regulatory cells (45). Therefore, differing levels of IL-2 in the priming microenvironment present in OVA/alum and IAV may contribute to regulating the differential generation of CXCR5^+^CD8^+^ T cells. However, further study is required to determine the factors that influence CXCR5^+^CD8^+^ T cell differentiation in diverse settings.

CXCR5^+^CD8^+^ T cells generated in chronic inflammatory settings are recognised as a distinct CD8^+^ T cell subset (46, 47). Here, we demonstrate that CXCR5^+^CD8^+^ T cells generated in response to both protein immunisation and IAV were enriched for early effector cells, exhibited lower expression of effector molecules and displayed a Tim-3^lo^TCF-1^hi^ signature which has been associated with CD8^+^ T cells that possess greater memory potential (48, 49). Further profiling revealed that CXCR5^+^CD8^+^ T cells display increased expression of Tfh-associated molecules including, ICOS, BTLA and Ly108, in both models. Indeed, more extensive characterisation of CXCR5^+^CD8^+^ T cells in LCMV infection, through RNA sequencing, revealed that they actively co-opt the follicular differentiation program and express a Tfh-associated gene signature, including increased expression of ICOS and Ly108 (11, 12). Thus, CXCR5^+^CD8^+^ T cells likely also co-opt the follicular differentiation program and localise toward B cell follicles following protein immunisation and IAV. Together, the results demonstrate that CXCR5^+^CD8^+^ T cells generated in response to protein immunisation and IAV represent a distinct, specialised population of effector CD8^+^ T cells and reflect the characteristics of CXCR5^+^CD8^+^ T cells identified early during chronic infections.

In line with exhibiting a less-exhausted effector cell phenotype with memory-like potential in response to protein immunisation and IAV, CXCR5^+^CD8^+^ T cells displayed limited cytotoxic potential as they expressed significantly reduced levels of the cytolytic molecule GzmB and demonstrated reduced *ex vivo* killing activity compared to their CXCR5^-^ counterparts. These results are consistent with previous findings on the cytotoxic capacity of CXCR5^+^CD8^+^ T cells in chronic LCMV (11). In contrast, He and colleagues (12) reported that CXCR5^+^CD8^+^ T cells in chronic LCMV possessed greater cytotoxic activity in an *in vivo* killing assay. Our data and the results from the study by Leong and colleagues are from experiments that assessed the cytotoxicity of CXCR5^+^CD8^+^ T cells relatively early, at days 7-8 post-immunisation/infection, while He and colleagues performed these analyses on cells obtained 21 days post-infection. Indeed, the profiling data from the study by He and colleagues also demonstrate that the CXCR5^+^CD8^+^ T cells isolated from later time points express more IFNγ and TNFα compared to their CXCR5^-^ counterparts, while they observed similar frequencies of cytokine-producing cells at day 8 post-infection, indicating both CXCR5^+^ and CXCR5^-^ CD8^+^ T cells late during chronic infection are phenotypically distinct from early cells and therefore likely differ functionally. At late time points during chronic infection, CXCR5^-^CD8^+^ T cells exhibit an exhausted CD8^+^ T cell phenotype while CXCR5^+^CD8^+^ T cells retain their proliferative and differentiation potential and can give rise to functional cytotoxic effector cells (13). These factors may account for the observed differences in cytotoxic activity between studies. Together, under the conditions tested, the results indicate that CXCR5^+^CD8^+^ T cells generated in response to protein immunisation and IAV have reduced cytotoxic potential, lending support to the notion of functionality outside of cytotoxicity in these settings.

Despite the majority of work exploring CXCR5^+^CD8^+^ T cell biology focusing on describing their cytotoxic roles during chronic inflammation in persistent infections and cancers, CD8^+^ T cells have been reported to localise within or directly adjacent to B cell areas of lymphoid tissues (50), yet the significance of this phenomenon is not understood. Intriguingly, a seminal study in 2007 describing CXCR5^+^CD8^+^ T cells in human tonsils, identified their capacity to support B cell survival and antibody production *in vitro* (10). Additional corroborating reports have found that CXCR5^+^CD8^+^ T cells can provide help for B cells in *in vitro* co-cultures (17–19). Furthermore, in settings where CXCR5^+^CD8^+^ T cell differentiation is exacerbated through interrupting IL-2 signalling, there is an associated increase in autoimmune antibody responses, indicating that CXCR5^+^CD8^+^ T cells can also promote self-reactive humoral immune responses (20, 21). In addition to their B cell helper activity, there are also reports of a specific population of Qa-1-restricted CD8^+^ T cells that adopt a follicular T cell phenotype, marked by CXCR5 expression, localise to B cell follicles and suppress autoreactive GCs through engaging and inhibiting Tfh cells in a Qa-1-dependent manner (42). Thus, the function of CXCR5^+^CD8^+^ T cells in humoral immunity is complex and appears to be highly context-dependent.

Here, we demonstrate that in response to protein immunisation and peripheral viral infection, CXCR5^+^CD8^+^ T cells shape the antibody response *in vivo*. CXCR5 expression is required on CD8^+^ T cells to promote class switching to IgG2c in responding B cells, likely through CXCR5-mediated local delivery of IFNγ to activated B cells at the T-B border. Indeed, our data show that the CXCR5^+^CD8^+^ T cell-mediated IgG2c class switching in the ASC compartment in OVA/alum is independent of changes in IFNγ secretion by responding CD4^+^ T cells and we also did not observe any changes in the total number of ASCs. This is in contrast to previous reports which proposed that these effects were indirect, where OT-I cells amplify IFNγ production in the CD4^+^ T cell compartment during the response to OVA/alum, leading to increased IgG2c-switching in the ASC compartment (24). In the present study, 10-fold fewer OT-I cells were adoptively transferred and there was no cotransfer of CD4^+^ OT-II cells, technical differences which may account for the different outcomes. Interestingly, when production of IFNγ is restricted to transferred CD8^+^ T cells (OT-I), CXCR5^+^CD8^+^ T cells also promote class switching to IgG2c in both the early GC and ASC compartments in response to IAV. Recently, Roco and colleagues reported that class switching events in B cells occur predominantly outside of the GC, in early antigen-activated B cells at the T-B border (51). Therefore, CXCR5^+^CD8^+^ T cells likely shape class switching in early activated B cells at the T-B border, while they may also contribute to regulating this process in committed GCB cells. Together, the results demonstrate that CXCR5^+^CD8^+^ T cells shape antibody responses *in vivo*, extending *in vitro* findings that indicate CXCR5^+^CD8^+^ T cells support B cell survival and antibody production (10, 17–21). However, further investigations are required to determine the mechanisms involved in communication between CXCR5^+^CD8^+^ T cells and B cells.

An important outstanding question from this study is where CXCR5^+^CD8^+^ T cells generated in response to protein immunisation and IAV are localising within the secondary lymphoid tissue microenvironment. Indeed, CXCR5 is well recognised for mediating immune cell homing to B cell follicles (4, 52, 53). CD4^+^ T cells are known to upregulate CXCR5 during the early stages of activation following immunisation (4, 54), and this is critical for directing their migration to the T-B border and B cell follicles (4). CXCR5 has also been implicated in directing follicular migration of CD8^+^ T cells. Elegant *in situ* imaging experiments tracking the endogenous tetramer-specific CD8^+^ T cell response to Listeria monocytogenes (Lm)-OVA observed antigen-specific CD8^+^ T cells localising to the T-B border during the response (50). In support of these observations, a recent study found that OT-I cells responding to Lm-OVA upregulate CXCR5 and localise to the T-B border (55). Furthermore, studies in the LCMV model comparing follicular-homing of WT and *Cxcr5^-/-^* (11) or sort-transferred CXCR5^+^ and CXCR5^-^ CD8^+^ T cells (12) have demonstrated that CD8^+^ T cells require CXCR5 expression to migrate to B cell follicles. In contrast to these reports detailing the essential role of CXCR5 in directing the follicular-homing of T cells, recent studies have demonstrated that CD4^+^ regulatory and helper T cell populations can localise to B cell follicles independently of CXCR5 expression (56, 57). Vanderleyden and colleagues found that CXCR5-deficient T follicular regulatory cells could still infiltrate GCs and B cell follicles, although their ability to do so was impaired in the absence of CXCR5 (56). Moreover, Greczmiel and colleagues demonstrated that CXCR5-deficient Tfh cells can migrate to GCs and B cell follicles, although the polarity of their distribution within the GC niche is disrupted in the absence of CXCR5 (57). Thus, the role of CXCR5 in directing follicular-homing of T cells appears to be context dependent. As CXCR5^+^CD8^+^ T cells have been shown to utilise CXCR5 to localise to B cell follicles in response to LCMV infection (11, 12), it is possible they also employ CXCR5-directed migration to localise to the follicular microenvironment during the response to protein immunisation and IAV infection. However, imaging studies are required to determine the positioning of CXCR5^+^CD8^+^ T cells generated in the latter contexts.

While we have demonstrated that CXCR5^+^CD8^+^ T cells influence physiological B cell responses in the secondary lymphoid tissue microenvironment of the lymph node, CD8^+^ T cells are known to support B cells in ectopic sites. Of note, Yang and colleagues reported that a population of IL-6-stimulated CD8^+^ T cells can provide IL-21 to support antibody production and ectopic B cell responses in inducible bronchus associated lymphoid tissue (iBALT) following IAV infection (58). IL-21 production by CD8^+^ T cells was restricted to the IL-6-rich lung microenvironment and absent in CD8^+^ T cells from the spleen and lymph nodes. Additionally, CD8^+^ T cells were found in close contact with GCB cells within iBALT at 14 days post infection (58). Furthermore, CD40L-expressing CD8^+^ T cells have been detected in follicles of ectopic lymphoid tissue in the synovium from patients with rheumatoid synovitis and their numbers positively correlated with the presence of ectopic GCs (59). However, these reports did not determine whether CXCR5 expression was a feature of IL-21- or CD40L-expressing CD8^+^ T cells. Together, these reports demonstrate that CD8^+^ T cells can support ectopic humoral immunity in both physiological and autoimmune settings. In the present study, we directly implicate CXCR5 expression by CD8^+^ T cells in their ability to shape class switching in physiological B cell responses within secondary lymphoid tissues, supporting the direct involvement of CXCR5^+^CD8^+^ T cells in autoimmune B cell responses (20, 21). Thus, it will be important to assess whether CXCR5^+^CD8^+^ T cells participate in ectopic response and to further determine how their role in humoral immunity may be manipulated to assist physiological responses while inhibiting their contributions to autoimmunity in both secondary and tertiary lymphoid tissues.

Overall, the findings from this study provide important insights into the context-specific biology of CXCR5^+^CD8^+^ T cells. Firstly, we show that CXCR5^+^CD8^+^ T cells are generated *in vivo* in acute responses to protein immunisation and peripheral viral infection, settings where there is no infection in the follicular microenvironment. In these contexts, CXCR5^+^CD8^+^ T cells display Tfh-like characteristics, indicative of co-option of the follicular differentiation pathway, exhibit reduced cytotoxic potential and possess a precursor-exhausted phenotype, features that also reflect the biology of CXCR5^+^CD8^+^ T cells identified in chronic settings in response to LCMV. Secondly, in addition to their described cytotoxic roles, we demonstrate that CXCR5^+^CD8^+^ T cells influence humoral immunity, shaping the antibody response *in vivo* following protein immunisation and peripheral viral infection, promoting class switching to IgG2c in responding B cells, a process dependent on expression of CXCR5. CXCR5^+^CD8^+^ T cell-mediated shaping of humoral immunity may impart the antibody response with the required class switched repertoire necessary for effective host defence, particularly where there is limited CD4^+^ T cell capability, such as that observed in later stages of HIV infection.

## Data Availability Statement

The raw data supporting the conclusions of this article will be made available by the authors, without undue reservation.

## Ethics Statement

The animal study was reviewed and approved by Animal Ethics Committee, University of Adelaide.

## Author Contributions

TT and KF contributed to the project conceptualisation, designed and performed experiments and wrote the manuscript. TN, EK, DM, and SD performed experiments. MA and DY provided important intellectual input and edited the manuscript. IC and SM conceptualised the project, designed experiments, supervised the study and wrote the manuscript. SM, IC, and MA obtained funding for the project. All authors contributed to the article and approved the submitted version.

## Funding

This research was supported by an NHMRC project grant 1163335 to SM, IC, and MA, and Research Training Program Scholarships to TT, KF, TN, EK, DM, and SD.

## Conflict of Interest

The authors declare that the research was conducted in the absence of any commercial or financial relationships that could be construed as a potential conflict of interest.

## Supplementary Material

Supplementary Figure 1 Data relating to **Figures 2–5**. **(A)** Gating strategy used to define TFH (CXCR5^hi^PD-1^hi^) and Pre-TFH (CXCR5^mid^PD-1^mid^) populations in the spleen in response to OVA-alum (left) and the mLN in response to x31-OVA (right). **(B-H)** Congenically marked WT (CD45.1/2) and *Cxcr5* ^-/-^ (CD45.2) OT-I cells (or PBS only for no transfer controls) were transferred i.v into separate B6.Ly5.1 (CD45.1) mice that were subsequently immunised with OVA/alum i.p and harvested on day 7 post-immunisation for analysis. **(B)** Frequency, **(C)** total number and **(D)** proportion expressing IFNγ following re-stimulation of TFH cells. **(E)** Proportion and **(F)** number of Th1 cells following re-stimulation. **(G)** Total number and **(H)** proportion expressing IFNγ of WT and *Cxcr5* ^-/-^ OT-I cells. **(I-K)** C57BL/6 mice were treated i.p. with either αCD8β or control antibody 4 days before and on day 4 and 12 after i.n infection with x31 and harvested on day 14 post-infection. **(I)** Levels of serum x31-specific IgG2c assessed by ELISA. **(J)** Total number of GCB cells and **(K)** number of IgG2c^+^ GCB cells in the mLNs of mice. **(L-N)** WT or *Cxcr5* ^-/-^ OT-I cells (or PBS only for no transfer controls) were transferred i.v. into separate B6.*Ifng^-/-^* mice that were then infected with x31-OVA i.n. **(L)** Gating strategy used to identify IFNγ-producing CD8^+^ T cells (OT-I) following re-stimulation. **(M)** Frequency and **(N)** total number of IFNγ-producing CD8^+^ T cells (OT-I). **(B-F)** Data are pooled from two independent experiments with a total of 10-12 mice per group. **(G)** Data are pooled from two independent experiments with a total of 8-9 mice per group. **(H)** Data are representative of two independent experiments, 4 mice per group. **(I-K)** Data are pooled from two independent experiments with a total of 9 mice per group and the absorbance values for the ELISA data have been normalised to the average of the Ctrl group for each independent experiment prior to pooling the data. **(L-N)** Data are representative of two independent experiments, 4-5 mice per group. **(B-F)** Data were analysed by ordinary one-way ANOVA, **(G, H, J, K, M, N)** unpaired t-tests or **(I)** two-way ANOVA with Bonferroni’s multiple comparison test. **(B-H, J, K, M, N)** Mean ± SEM, **(I)** Mean + SD.
